# LysoPC acyltransferase/PC transacylase activities in plant plasma membrane and plasma membrane-associated endoplasmic reticulum

**DOI:** 10.1186/1471-2229-7-64

**Published:** 2007-11-28

**Authors:** Karin E Larsson, J Magnus Kjellberg, Henrik Tjellström, Anna Stina Sandelius

**Affiliations:** 1 Department of Plant and Environmental Sciences, Göteborg University, P.O. Box 461, SE-405 30 Göteborg, Sweden; 2 Lead Discovery Informatics Team, Lead Generation Department, AstraZeneca R&D Mölndal, Sweden

## Abstract

**Background:**

The phospholipids of the plant plasma membrane are synthesized in the endoplasmic reticulum (ER). The majority of these lipids reach the plasma membrane independently of the secretory vesicular pathway. Phospholipid delivery to the mitochondria and chloroplasts of plant cells also bypasses the secretory pathway and here it has been proposed that lysophospholipids are transported at contact sites between specific regions of the ER and the respective organelle, followed by lysophospholipid acylation in the target organelle. To test the hypothesis that a corresponding mechanism operates to transport phospholipids to the plasma membrane outside the secretory pathway, we investigated whether lysolipid acylation occurs also in the plant plasma membrane and whether this membrane, like the chloroplasts and mitochondria, is in close contact with the ER.

**Results:**

The plant plasma membrane readily incorporated the acyl chain of acyl-CoA into phospholipids. Oleic acid was preferred over palmitic acid as substrate and acyl incorporation occurred predominantly into phosphatidylcholine (PC). Phospholipase A_2 _stimulated the reaction, as did exogenous lysoPC when administered in above critical micellar concentrations. AgNO_3 _was inhibitory. The lysophospholipid acylation reaction was higher in a membrane fraction that could be washed off the isolated plasma membranes after repeated freezing and thawing cycles in a medium with lowered pH. This fraction exhibited several ER-like characteristics. When plasma membranes isolated from transgenic *Arabidopsis *expressing green fluorescent protein in the ER lumen were observed by confocal microscopy, membranes of ER origin were associated with the isolated plasma membranes.

**Conclusion:**

We conclude that a lysoPC acylation activity is associated with plant plasma membranes and cannot exclude a PC transacylase activity. It is highly plausible that the enzyme(s) resides in a fraction of the ER, closely associated with the plasma membrane, or in both. We suggest that this fraction might be the equivalent of the mitochondria associated membrane of ER origin that delivers phospholipids to the mitochondria, and to the recently isolated ER-derived membrane fraction that is in close contact with chloroplasts. The *in situ *function of the lysoPC acylation/PC transacylase activity is unknown, but involvement in lipid delivery from the ER to the plasma membrane is suggested.

## Background

The composition of the lipid phase of plant plasma membranes adjusts to the varying conditions in the plant environment. The adjustments include selective lipid degradation, increased incorporation of certain lipid classes and/or lipid molecular species and possibly re-tailoring of the lipids within the membrane as well [1-5]. In addition to their structural role, plasma membrane lipids are crucial intermediates in several signaling pathways [6].

*De novo *synthesis of plasma membrane phospholipids occurs mainly in the endoplasmic reticulum (ER) [7-9]. The major plasma membrane phospholipids, phosphatidylcholine (PC) and phosphatidylethanolamine (PE) with C_16 _and C_18 _acylation of the *sn-1 *and *sn-2 *positions of the glycerol backbone, respectively, have been reported to be transported to the plasma membrane independently of the vesicular secretory pathway [8,9]. The nature of lipid transport to the plant plasma membrane outside this pathway remains to be established, but for yeast and/or animal cells, lipid transport has been demonstrated to occur at membrane contacts sites (MCSs) between for example ER and mitochondria and ER and trans-Golgi membranes [10,11]. In yeast, a plasma membrane-associated ER region was isolated. The fraction was denoted PAM (plasma membrane associated membrane), and lipid synthesis was enriched compared to bulk ER, whereas transport of lipids remains to be demonstrated [12]. MCSs between ER and plasma membranes have not been reported for plants, but a close proximity between these membranes has been visualized by freeze fracture microscopy of suspension-cultured sycamore cells [13] and by confocal microscopy of *Arabidopsis *transformed with fluorescent tags on specific ER membrane proteins [14].

Mitochondria and chloroplast PC are also of ER origin [8]. Presently, the most favoured model for lipid delivery to the mitochondria is that of lipid delivery at contact zones between a specialized ER region, denoted MAMs (mitochondria associated membranes), and the mitochondria [15]. Biochemical [16-19] as well as biophysical [20] evidence is emerging for corresponding zones of contact between chloroplasts and a special region of the ER, denoted PLAMs (plastid associated membranes). Mitochondria [21] and chloroplasts [16,18,19] isolated from plant tissue both possess highly active lysoPC acylation activities and it has been suggested that in both cases, lysoPC is the lipid transported from the closely associated ER to the respective organelle.

To investigate the possibilities that phospholipid delivery to the plant plasma membrane outside the secretory apparatus could involve acylation of transported lysophospholipid and that a region of the ER could be involved, analogous to the situation for mitochondria and chloroplasts, we examined lysophosholipid acylation in isolated plasma membrane and a putative PAM fraction. We also present evidence for a PAM fraction in association with the plasma membrane.

## Results

### Membrane fractionation

**Figure 1:**
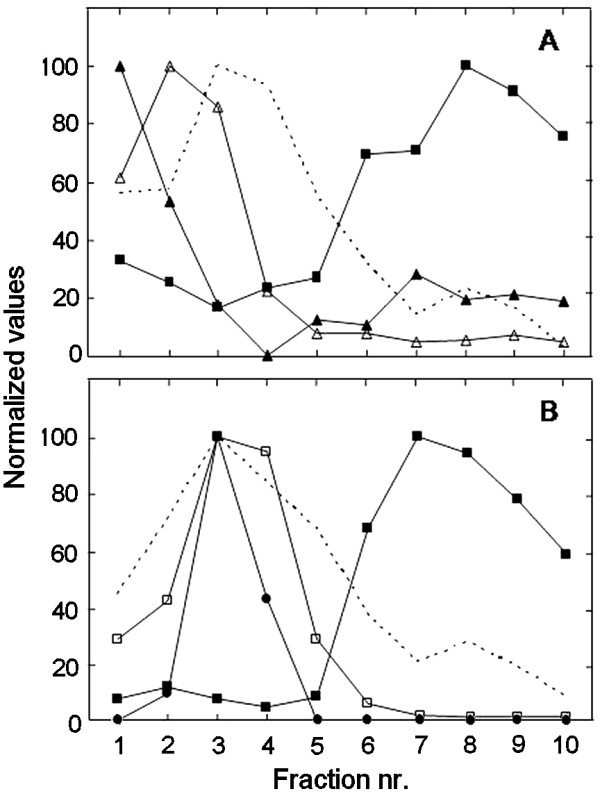
**The distribution of protein and enzyme activities in membrane fractions obtained from pea seedlings**. A microsomal membrane fraction was fractionated by a 10-step aqueous polymer two-phase counter current distribution and the resulting 10 membrane fractions analyzed. **A**, The normalized activities of choline phosphotransferase for ER (solid triangles; 100 corresponds to 10 pmol·[mg protein]^-1^·min^-1^), cytochrome C oxidase for mitochondrial inner membrane (open triangles; 100 corresponds to 1.46 mmol·[mg protein]^-1^·min^-1^) and 1,3-β-glucan synthase for plasma membrane (solid squares; 100 corresponds to 0.53 μmol·[mg protein]^-1^·min^-1^). The dashed line shows the normalized protein distribution between the fractions. **B**, The normalized chlorophyll content of thylakoid (open squares; 100 corresponds to 0.12 mg·[mg protein]^-1^), the normalized binding of a monoclonal anti-β-COP anti body for Golgi (solid circles; antibody binding only occurred to fractions 2–4, 100 corresponds to the highest binding), 1,3-β-glucan synthase for plasma membrane (solid squares; 100 corresponds to 0.43 μmol·[mg protein]^-1^·min^-1^). and the normalized distribution of protein between the fractions (dashed line). As all parameters could not be assayed on the fractions from a single 10-step membrane distribution, the panels A and B present the results from two independent experiments, with the plasma membrane marker and the protein distribution analyzed for both.

**Figure 2:**
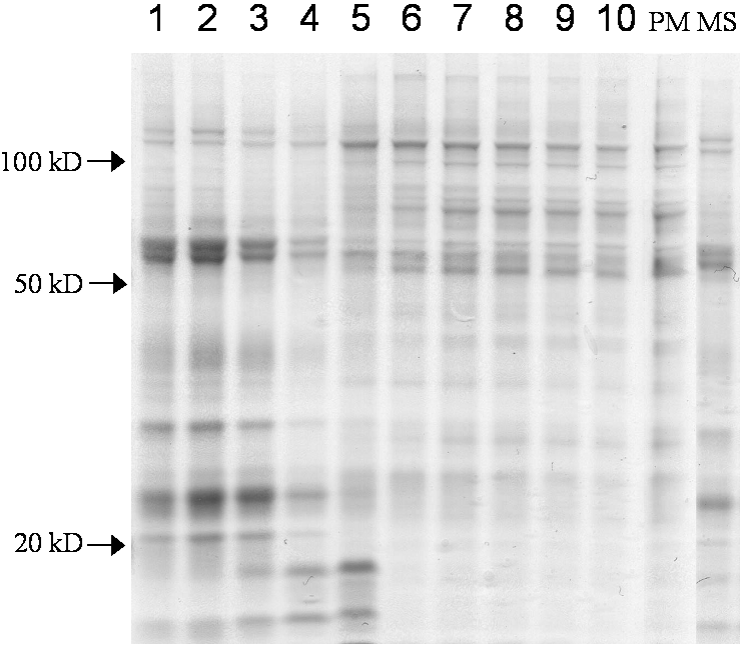
**Polypeptide patterns of pea membrane fractions**. Comassie Brilliant Blue-stained SDS gels are shown for separated polypeptides from membrane fractions obtained from a 10-step aqueous polymer two phase counter current distribution of pea seedling microsome membranes (1–10; cf. Fig. 1), pea plasma membrane (PM) and pea microsome membranes (MS). The arrows mark the positions of molecular weight markers.

The purities of the plasma membrane fractions had been established previously for both pea (traces of ER and chlorophyll only [22]) and soybean (95% plasma membrane, as judged by morphometry after phosphotungstic acid staining at low pH of thin sections for electron microscopy [23]). Renewed marker enzyme assays verified the purities of the isolated fractions (results not shown). For pea, we assayed marker enzyme activities also on membrane fractions obtained from fractionation of shoot microsomal membranes by a 10-step aqueous polymer two-phase counter current distribution [24]. Figure 1 shows the distribution of proteins and of markers for mitochondrial inner membranes, ER, Golgi apparatus, thylakoids and plasma membranes between the 10 fractions. Mitochondrial inner membranes, Golgi membranes and thylakoids were recovered in the earlier fractions, whereas plasma membranes were recovered predominantly in fractions 6–10. The ER marker choline phosphotransferase was predominantly recovered in the first two fractions and a minor second peak co-localized with the plasma membrane marker, in fractions 6–10. The polypeptide patterns of the fractions reflected the marker assays, with fractions 6–10 being remarkably similar to that of isolated plasma membrane (Fig. 2). The prominent > 100 kD band of fractions 6–10 and the plasma membrane probably represented the P-type ATPase, whereas the prominent 55–60 kD bands in fractions 1–3 probably represented the mitochondrial ATP synthase α- and β-chains and ADP/ATP transporter, respectively [24]. The differences in intensity of these bands in fractions 1–4 followed the differences in specific activity of the mitochondrial marker enzyme in these fractions (cf. Figures 1 and 2).

### Plasma membrane lipid acylation

**Figure 3:**
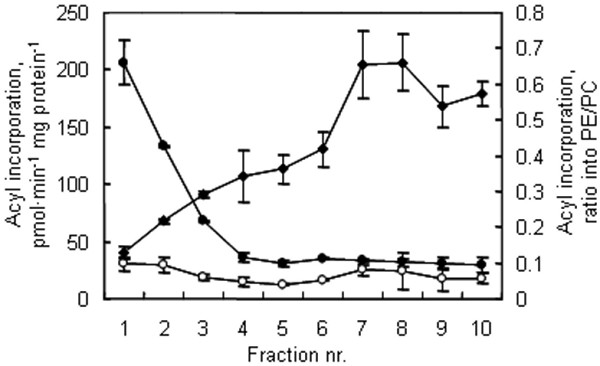
**The lipid acylation activities in pea seedling membrane fractions**. A microsomal membrane fraction was fractionated by a 10-step aqueous polymer two-phase counter current distribution and the resulting membrane fractions were incubated with [^14^C]18:1-CoA to monitor acyl incorporation into phospholipids. Filled circles, acyl incorporation into PC; open circles, acyl incorporation into PE; filled diamonds, the PE/PC ratio of acyl incorporation.

Incorporation of [1-^14^C]18:1-CoA into native PC and PE was analysed in the membrane fractions obtained from the 10-step counter current two-phase separation. The incorporation of [1-^14^C]18:1-CoA into PC occurred in all 10 fractions, but was markedly strongest in the first two fractions (Fig. 3), thus co-migrating with the ER membrane marker choline phosphotransferase (cf. Fig. 1A). The result is consistent with the model that the plant ER contains a highly active lysoPC acyl transferase [25,26]. Acyl group incorporation into PE also occurred in all fractions, with similar rates in the ER- and plasma membrane-containing fractions. In the plasma membrane fractions (cf. Figs. 1A and 3) the ratio of acyl group incorporation into PE over PC was the highest, between 0.4–0.7.

**Figure 4:**
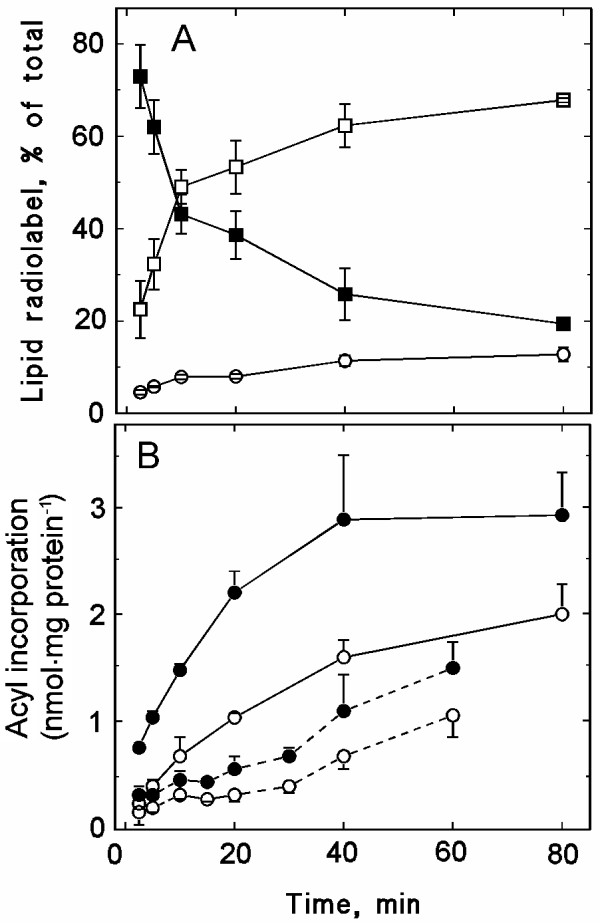
**Time dependence of the incorporation of radiolabel from [^14^C]acyl-CoA into isolated plasma membranes**. Plasma membranes, isolated from pea seedlings, were incubated with radiolabelled acyl-CoA for up to 80 min. Each incubation contained 25 μg plasma membrane protein. **A**, Distribution of radiolabel between 18:1-CoA (solid squares), free fatty acids (open squares) and phospholipids (open circles). **B**, Incorporation of radiolabel into PC (filled symbols) and PE (open symbols). Either [^14^C]18:1-CoA (solid line) or [^14^C]16:0-CoA (dashed line) was used as substrate. The data represent mean values ± the range of duplicates from a representative experiment.

To investigate lipid acylation in more detail, we used isolated plasma membrane fractions. When pea plasma membranes were incubated with [1-^14^C]18:1-CoA (Fig. 4A) or [1-^14^C]16:0-CoA (results not shown), the radiolabel was recovered predominantly as free fatty acids, indicating that the plasma membranes contained a highly active acyl-CoA thioesterase. As acyl-CoAs play important roles in cellular processes such as regulation of enzyme activities, membrane fusion and signal transduction [27], the high thioesterase activity might reflect that the plasma membrane has the capability to regulate the size of the acyl-CoA pool in its vicinity.

The remaining acyl-CoA radiolabel was recovered in phospholipids, predominantly PC (Fig. 4B). The labelling was higher with [1-^14^C]18:1-CoA than with [1-^14^C]16:0-CoA as substrate, indicating a preference for acyl incorporation into the *sn-2 *position. After 30 min incubation with [1-^14^C]18:1-CoA, 62% of the phospholipid radiolabel was recovered in PC, whereas 27 and 11% was recovered in PE and phosphatidylinositol (PI), respectively (results not shown). With [1-^14^C]16:0-CoA, acyl incorporation was more evenly distributed between these three lipid classes: after 30 min incubation 39, 35 and 26% of the phospholipid radiolabel was recovered in PC, PE and PI, respectively (results not shown). Radiolabel was never recovered in phosphatidylglycerol. However, in a few experiments, 2–5% of the acyl-CoA radiolabel was recovered in phosphatidic acid (PA) (results not shown). When the radiolabelled acyl-CoA substrates were substituted with the corresponding radiolabelled free fatty acids, no radiolabelling of phospholipids was observed, demonstrating that the substrate for the acyltransferase was acyl-CoA (results not shown).

**Figure 5:**
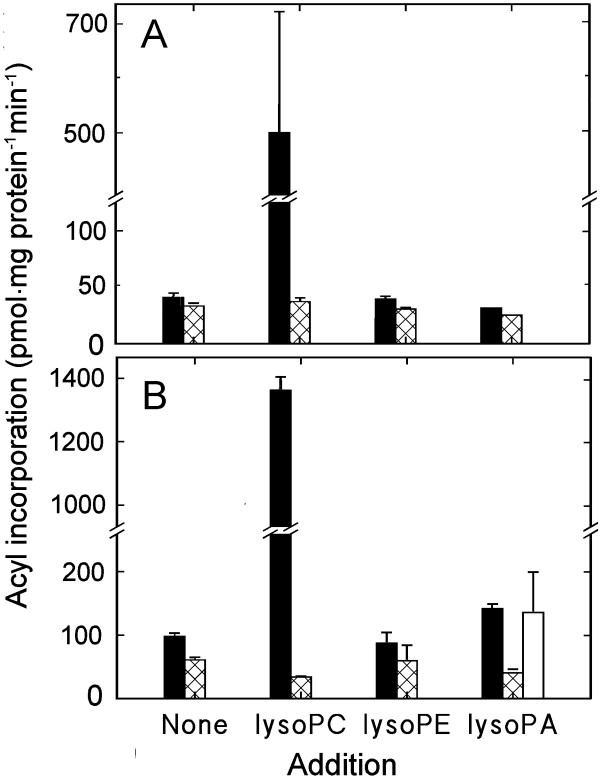
**The effects of lysophospholipids on the incorporation of radiolabel from [^14^C]acyl-CoA into plasma membrane phospholipids**. Plasma membranes were isolated from pea seedlings and incubated for 30 min with either [^14^C]16:0-CoA (**A**) or [^14^C]18:1-CoA (**B**). The concentration of added lysophospholipid was 70 μM (lysoPC and lysoPE were predominantly acylated with 16:0 or 18:0, lysoPA was acylated with 18:1). Radiolabel recovery is presented for PC (solid bars), PE (cross-hatched bars) and PA (open bars). Otherwise as in Figure 4.

**Figure 6:**
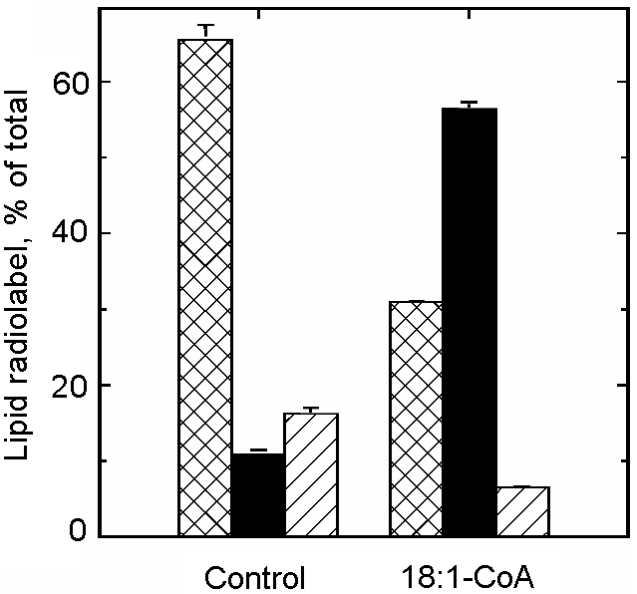
**Metabolism of exogenous lysoPC in isolated plasma membranes**. Plasma membranes were isolated from hypocotyls of dark-grown soybean and incubated with 80 μM *sn-1 *[^14^C]16:0-lysoPC/egg yolk PC (0.22 GBq/mmol) for 30 min in the absence (control) or presence of 100 μM non-radiolabelled 18:1-CoA. The distribution of radiolabel between lysoPC (cross-hatched bar), PC (solid bar) and free fatty acids (diagonally hatched bar) is based on the data from one representative experiment and mean values and the range of duplicates are presented.

The incorporation of radiolabel from acyl-CoA into a phospholipid could reflect acylation of the corresponding lysophospholipid and/or re-tailoring of the phospholipid. To investigate whether lysolipids functioned as substrate, two approaches were used. In the first approach, different amounts of lysoPC, lysoPE and lysoPA were included in the assay, together with radiolabelled acyl-CoA. At concentrations slightly below the critical micellar concentration (cmc; 7 μM for C_16_-lysoPC, lower for lysoPE; [28]), no increase in radiolabel incorporation into phospholipids was detected (results not shown). When 70 μM lysoPC was included, there was a drastic increase in the labelling of PC both with [1-^14^C]16:0-CoA (Fig. 5) and [1-^14^C]18:1-CoA (Fig. 5, Table 1). The same concentration of lysoPA stimulated labelling of PA but also of PC. This stimulation was markedly smaller and occurred only with [1-^14^C]18:1-CoA. Labelling of PE remained unaffected by inclusion of 70 μM of its lyso derivative. When plasma membranes were incubated with radiolabelled lysoPC and non-radiolabelled acyl-CoA, radiolabelled PC was formed, verifying the reaction (Fig. 6).

**Table 1:** Acyl incorporation into PC and PE, comparing PAM with plasma membranes and an ER-rich fraction

	Acyl incorporation from [^14^C]18:1-CoA (pmol·min^-1^·[mg protein]^-1^)	
	
	PC	PE
Plasma membrane		
on its own	53	23
+ lysoPC	549	47
+lysoPC + AgNO_3_	7	6
PAM-decreased plasma membrane		
on its own	89	27
+ lysoPC	329	37
+ lysoPC + AgNO_3_	11	5
PAM		
on its own	74	20
+ lysoPC	840	49
+ lysoPC + AgNO_3_	14	10
Enriched ER		
on its own	91	13
+ lysoPC	899	53
+ lysoPC + AgNO_3_	15	8

A pea shoot plasma membrane fraction was the starting material for the isolation of the PAM fraction. The PAM-decreased plasma membrane fraction represents the plasma membranes obtained after the removal of the PAM. The ER-enriched fraction is fraction number 1 from the 10-step counter current distribution (see Fig. 1). All fractions were incubated with [^14^C]18:1-CoA on its or in the presence of 70 μM lysoPC (acylated with 18:1), without or with 80 μM Ag NO_3_. Mean values are presented for 2–5 independent experiments, with standard deviations less than 10%.

**Figure 7:**
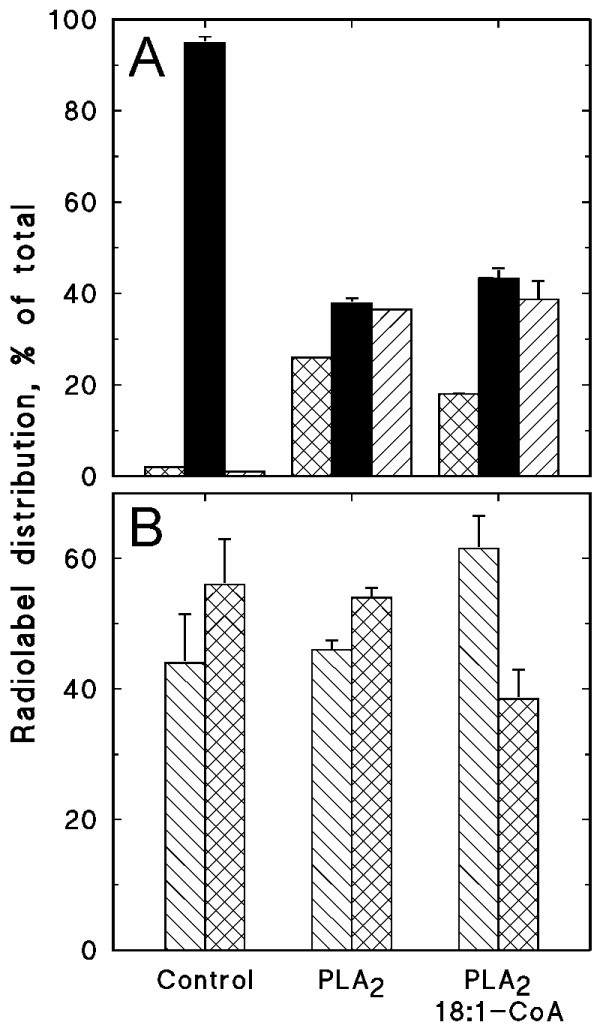
**The effects of phospholipase A_2 _(PLA_2_) and 18:1-CoA on the metabolism of exogenous PC in isolated plasma membranes**. Plasma membranes, isolated from hypocotyls of dark-grown soybean, were incubated with 0.25 μM *sn-1,2*-[^14^C]18:1-PC (3.7 GBq/mmol) in buffer containing 0.05% (w/v) Triton X-100 for 30 min, without or with the indicated additions (0.001 U bee venom PLA_2_, 100 μM non-radiolabelled 18:1-CoA). **A**, The distribution of radiolabel between lysoPC (cross-hatched bar), PC (solid bar) and free fatty acids (diagonally hatched bar). **B**, The distribution of radiolabel between the *sn*-1 (diagonally hatched bar) and *sn*-2 (cross-hatched bar) positions of [^14^C]PC. The data represent the mean values and range from duplicates.

The second approach to study the substrate role of lysophospholipids was to investigate whether stimulated formation of these lipids in the membrane had any effects. Endogenous PLA_2 _activity was low against exogenously supplied radiolabelled PC, but exogenous PLA_2 _stimulated the formation of lysoPC and free fatty acids from this substrate (Fig. 7A). When non-radiolabelled 18:1-CoA was included in the assay, the proportion of radiolabel associated with lysoPC decreased whereas that of PC increased (Fig. 7A). The resulting PC pool exhibited a decreased radiolabelling of the *sn*-2 position, demonstrating that both the PLA_2 _and re-acylation activities occurred at the *sn*-2 position (Fig. 7B). As bee venom PLA_2 _supposedly acts rather un-specifically on membrane phospholipids [29], an increase in several lysophospholipid classes would be the expected result. However, concomitant incubation of plasma membranes with PLA_2 _and radiolabelled acyl-CoA, resulted in increased radiolabelling of PC only, and only with [1-^14^C]18:1-CoA as substrate (results not shown), which suggests that the lipase was more specific towards PC than generally assumed or that the lysolipid acylase strongly preferred lysoPC.

### Lipid acylation in PAM, a fraction of plasma membrane-associated membranes

The 10-step counter current membrane fractionation revealed that the ER marker choline phosphotransferase was active in fractions enriched in plasma membrane (Fig. 1A), indicating presence also of ER in these fractions. With isolated pea plasma membranes as starting material, we isolated a light membrane fraction, denoted PAM. We investigated acyl incorporation into PC and PE in the starting plasma membrane fraction, the PAM fraction, the PAM-free plasma membranes and the first, ER-rich, fraction from the 10-step counter current procedure. Acyl group incorporation from [1-^14^C]18:1-CoA into PC and PE verified the earlier results, that acyl incorporation into PC was faster in the ER-rich fraction than in the plasma membrane, whereas the reverse applied for acyl incorporation into PE (Table 1). Acyl incorporation into PC appeared somewhat sensitive to the procedure employed to fractionate the plasma membrane fraction, as this activity were lower in the PAM and PAM-free plasma membranes. Addition of lysoPC markedly stimulated acyl incorporation into PC in all fractions, with the highest rates in the ER-rich and PAM fractions. Here, the plasma membrane fraction was intermediate between the PAM and PAM-free plasma membrane fractions. The lysoPC acylation activity was sensitive to AgNO_3_, a known inhibitor of lysoPC acyl transferase [30], suggesting that a major part of the observed lysoPC acylation was indeed catalysed by this transferase.

### Plasma membrane and PAM polypeptides

We separated the pea plasma membrane polypeptides by native gel electrophoresis and analyzed excised 1 mm sections for lysoPC acylation. The activity was present as a broad peak co-migrating with a broader major protein peak (results not shown). The fraction with the highest lysoPC acylation activity was submitted to tryptic digestion and linear ion trap mass spectrometry. The fraction contained a large number of polypeptides, but none related to lipid metabolism, probably due to the lack of annotation not only of pea peptides but also largely of lipid-related proteins.

**Figure 8:**
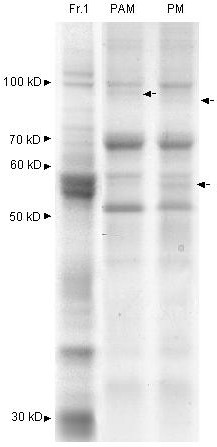
**A comparison of the polypeptide pattern of a PAM fraction with that of plasma membranes and an ER-rich fraction**. The polypeptide patterns are shown for an ER-rich membrane fraction (Fr. 1, which is identical to the first fraction of the separated pea microsomes presented in Figure 1), a fraction of plasma membrane associated membranes (PAM; obtained from a plasma membrane fraction) and isolated plasma membrane (PM). The arrows to the right of the lanes point to differences between the PAM and PM fractions. Otherwise as in Fig. 2.

The PAM fraction polypeptide pattern was very similar to that of the plasma membrane, but certain polypeptide bands were specific for one of the fractions only (Fig. 8, arrows). Analysis of the specific PAM polypepetide bands as above has so far failed to yield any information as to polypeptide identity.

### Optical evidence for PAMs

**Figure 9:**
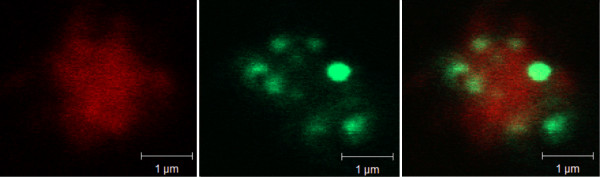
**Optical evidence for PAMs**. Confocal laser beam microscopy of a plasma membrane fraction isolated from 2 month-old *Arabidopsis thaliana*, transformed with green fluorescent protein (GFP) tagged to the ER lumen [31]. The isolated plasma membranes were subjected to a repeated cycle of freezing and thawing to invert a population of the vesicles [32] and incubated with the red fluorescent probe FM4-64. The sample was observed under a 488 nm laser which excited the plasma membrane-localized probe (left image) and under a 543 nm laser which excited ER-localized GFP (middle image). To the right, these two images are merged.

Using transformed *A. thaliana*, tagged with green fluorescent protein (GFP) in the ER lumen [31], we previously showed that the ER forms a network throughout the cytosol and that small green fluorescing bodies were associated with isolated chloroplasts [20]. Plasma membranes were isolated from the leaves of this transformed *A. thaliana *and subjected to repeated cycles of freezing and thawing to invert a population of the vesicles [32]. When a concentrated suspension of these plasma membranes was incubated with a fluorescent probe for membrane lipids, FM4-64, and observed by confocal laser beam microscopy, the membranes appeared red (Fig 9, left panel). Small fluorescent green bodies were also evident (Fig. 9, middle panel) and when these images were merged, it was evident that the ER-derived GFP-fluorescing bodies co-localized with the plasma membranes (Fig. 9, right panel). FM4-64 apparently had a preference for plasma membrane over ER, as evidenced from e.g. the lower right corner in the images, where the fluorescence of FM4-64 and GFP did not overlap (Fig. 9). We observed that a portion of the plasma membrane material was not associated with green fluorescent bodies, but green fluorescent bodies never appeared on their own, only together with plasma membranes (results not shown).

## Discussion

Acylation of lysoPC has been demonstrated to occur in ER, chloroplasts and mitochondria, isolated from plant tissues. We here report that also plant plasma membranes catalyse the incorporation of acyl groups from acyl-CoA into phospholipids, with the dominant product being PC. The incorporation of the acyl group of acyl-CoA into plasma membrane phospholipids could have one or several roles: (i) to adjust the acylation pattern of plasma membrane phospholipids by trans-acylation as an involvement in the response to an altered external condition (stress), (ii) to acylate lysophopsholipids to turn off signalling systems that utilize PLA_2_, and/or (iii) to acylate lysophospholipids to provide the final step in supplying the plasma membrane with PC, analogous to that reported for mitochondria and chloroplasts, namely acylation of lysoPC of ER origin.

We clearly could demonstrate lysolipid acylation. The concentration of lysophospholipids in isolated plasma membranes has usually been reported to be very low [1-5], which fits well with their proposed signalling and regulatory roles. For example, lysoPC and lysoPE have been proposed to act as second messengers to auxin [33] and to be involved in systemic responses in wounded plants [34]. LysoPC was recently reported to induce host plant genes that are involved in arbuscular mycorrhizal symbiosis [35]. LysoPE and lysoPI have been shown to inhibit, but lysoPA to stimulate, plant phospholipase D [36]. Lysolipids also affect membrane fusion [37]. Evidently, the lysophospholipid content of the plasma membrane is strictly regulated. A lysophospholipid acylation activity may thus function to regulate signal-response cascades, enzyme activities or the fusibility of the plasma membrane.

Since 18:1-CoA was preferred over 16:0-CoA in the acylation reaction, the endogenous lysophospholipids probably had derived from PLA_2_-catalyzed phospholipid degradation. This conclusion is based on that plant plasma membrane phospholipids usually contain unsaturated C_18 _fatty acids in the *sn-2 *position, whereas 16:0 is usually restricted to the *sn*-1 position [38]. The acylation activity increased when the content of lysophospholipids increased through addition of exogenous PLA_2_. The activity of PLA_2 _has been shown to suddenly increase at a threshold concentration of added lysoPC [39]. This activation was thought to reflect an increased susceptibility of the membrane to PLA_2_. A similar change in membrane properties at a threshold concentration of lysophospholipid could be relevant also in the present case. However, the added lysoPC did not only stimulate the PLA_2 _activity, as incubations with radiolabelled lysoPC demonstrated their role as substrate for the acylation reaction.

When provided with exogenous lysophospholipid, the plasma membrane acylation activity had a markedly higher specificity for lysoPC than for other lysophospholipids. Larger than cmc concentrations of lysoPC was required to stimulate the reaction, which could reflect that the enzyme is activated *in situ *by a high local concentration of the substrate. The stimulatory effect of lysoPA on lysoPC acylation could indicate a regulatory role for lysoPA. The sensitivity to AgNO_3 _indicates that the activity is related to previously reported lysoPC acyl transferases.

Without added lysophospholipids, acyl group incorporation from acyl-CoA into phospholipids occurred with several phospholipid classes, whereas with exogenous substrate, only lysoPC was acylated. These results may suggest that more than one lysophospholipid acyl transferase is present in the plant plasma membrane. Another interpretation is that acyl group incorporation from acyl-CoA into phospholipids in the absence of added lysophospholipid represented a transacylase activity.

It has been demonstrated that PC and PE acylated with C_16_/C_18 _fatty acids are delivered to the plasma membrane independently of the vesicular secretory pathway [8,9]. As it has been demonstrated that ER closely associates with the plasma membrane in yeast [12] and plant cells [13], we hypothesized that the delivery of lysoPC to the plant plasma membrane could occur at regions in close contact with the ER. Support for the hypothesis comes from several sources. In the 10-step counter-current separation of pea shoot membranes, the ER marker choline phosphotransferase to a minor extent also migrated with the plasma membrane marker (Fig. 1). In a previous 10-step separation of root membranes from phosphate-deficient oat [24], the ER marker NADPH cytochrome C reductase exhibited a dual distribution with the minor peak co-migrating with the plasma membrane. In an earlier report of a 5-step counter-current separation of microsomal membranes from cauliflower inflorescences [40], NADPH cytochrome C reductase and choline phosphotransferase activities co-localized in one major peak in the first tube as well as in a minor one co-migrating with the plasma membrane marker. In addition, we also observed that when plasma membranes were isolated from leaves of *A. thaliana *tagged with GFP in the ER lumen, small ER-derived structures co-isolated with the plasma membranes (Fig. 9). The association between the membranes was strong enough to survive the repeated freezing- and thawing procedure employed to invert a portion of the plasma membrane vesicles.

To isolate a putative membrane fraction of ER origin from the isolated plasma membrane, we modified the method developed for yeast PAMs [12]. As aqueous polymer two-phase partition isolates cytoplasmic side-in plasma membrane vesicles [32], the putative PAM would be present inside the isolated plasma membrane vesicles. We therefore had to invert the plasma membrane vesicles prior to the yeast protocol treatment of lowering the pH to separate the PAMs from the plasma membrane [12]. The protein patterns of the PAM and plasma membrane fractions were largely similar, although some proteins of the plasma membrane fraction were missing from the PAM fraction, whereas others were slightly enriched in the PAM fraction. Apparently, the PAM fraction was contaminated with plasma membrane. The freezing and thawing procedure employed to invert the plasma membrane vesicles prior to the PAM isolation probably resulted in the formation of small plasma membrane vesicles that co-isolated with the light PAM membrane vesicles. In addition, the freezing and thawing process could also have produced vesicles with surface properties intermediate between cytosolic side-out and cytosolic side-in plasma membrane vesicles, as we earlier reported for wheat plasma membranes [41]. In the present case, we therefore cannot rule out that the isolation process could have produced vesicles of mixed plasma membrane and PAM origin.

In the PAM, addition of lysoPC caused a stronger increase in acyl group incorporation into PC compared with the original plasma membrane fraction, whereas in the PAM-decreased plasma membrane, the activity was much lower than in the original plasma membrane fraction. The differences may suggest different sets of acyl transferases or transacylases in the plasma membrane and its adjacent PAM. Another explanation could be that lysoPC acylation/PC transacylation actually resides in the ER regions associated with the plasma membrane, the PAM. If this is the case, the contamination of the PAM fraction with plasma membrane would have diluted the higher PAM-associated activity and as all PAM probably was not washed away from the plasma membrane, the difference in acylation/transacylation acitivies between the two fractions would have been underestimated. If lipid acylation/transacylation activities of isolated plasma membrane actually reflect PAM activities, such lipid metabolizing activities are not evenly distributed over the ER. A future extended characterization of PAM awaits development of an isolation protocol that increases fraction yield and purity.

We did not succeed in identifying any acyl transferase among the peptides present in the plasma membrane peptide fraction with the highest lysolipid acylation activity, which may reflect that these types of enzymes are not well characerized and therefore not annotated.

## Conclusion

Our results demonstrate that isolated plant plasma membranes possess phospholipid acylation and/or transacylation activities. With endogenous substrate, the activity had a different lysophospholipid substrate preference than the corresponding activity of the ER. With added lysophospholipid substrate, both fractions were highly specific for lysoPC. We also present visual evidence for and the first tentative isolation of a plant PAM fraction, a membrane fraction of putative ER origin closely associated with the plasma membrane. The lysoPC acyl transferase activity was higher in this PAM fraction than in its parent plasma membrane fraction, whereas it was markedly lowered in the PAM-decreased plasma membrane. We propose that the zones of close contact between the ER and the plasma membrane, the PAMs, represents areas of the ER specialized in providing the plasma membrane with precursor for as well as synthesis of the PC that is delivered to the plasma membrane outside the secretory vesicular pathway. Whether both lysoPC acyl transferase and/or PC transacylase are constituents of the plasma membrane or the former or both activities are restricted to PAM is not yet resolved. The transport route for PE remains elusive.

## Methods

### Plant material and chemicals

Garden pea (*Pisum sativum *L., var Kelvedon Wonder) [16], soybean (*Glycine max *[L.] Merr., var. Folke) [23] and *A. thaliana *[20] were cultivated for 9–10 days, 4 days and 4–5 weeks, respectively, as described. The seeds of the transformed *A. thaliana *were kindly provided by Prof. I. Hara-Nishimura [31].

All chemicals and organic solvents were of analytical grade. Lipid standards, polyethylene glycol (PEG) and fine chemicals were obtained from Sigma (St. Louis, MO, USA) and organic solvents, TLC plates and inorganic salts from Merck (Darmstadt, Germany). Radiochemicals and Dextran T-500 were from Amersham Pharmacia Biotech (Uppsala, Sweden), and the fluorescent probe FM4–64 was from Invitrogen, Molecular Probes (Eugene, OR, USA).

### Isolation of membrane fractions

Plasma membranes were isolated from microsomal fractions (filtered homogenate centrifuged for 10 min at 6 000 *g*_max_, microsomes pelleted from for 30 min at 65 000 *g*_max_) by aqueous polymer two-phase partitioning using one sample and two wash systems. The compositions of the systems were as previously optimized in the lab for pea shoots [22], soybean hypocotyls [23] and *A. thaliana*. The isolated plasma membranes were suspended in 0.25 M sucrose, 10 mM KCl, and 10 mM HEPES/KOH, pH 7.0, and used directly or frozen in liquid N_2 _prior to storage at -80°C.

To determine whether a membrane fraction of ER origin was associated with the plasma membranes, isolated pea shoot plasma membranes were suspended in 10 mM MES/KOH pH 6.0, and 0.33 M sucrose and subjected to three cycles of freezing in liquid nitrogen followed by thawing. As plasma membranes vesicles isolated by two phase partitioning are predominantly or exclusively oriented with the cytoplasmic surface inwards, this treatment was necessary to expose the cytoplasmic surface of a portion of the vesicles [32]. A lowered pH was used as it had been required to separate PAMs from isolated yeast plasma membrane [12]. After 10 min incubation at 4°C, the membrane suspension was top-loaded onto continuous sucrose gradient (20–50% w/v sucrose) in the same buffer. The gradient was centrifuged for 60 min in a swing out rotor at 100 000 × g_max_. The band at the top of the gradient was collected as plasma membrane associated membranes (PAMs). These membranes and the resuspended plasma membrane pellet were diluted with 30 mM HEPES/KOH pH 7.0, 10 mM KCl and 2.5 mM MgCl_2 _and pelleted at 100 000 × g_max _for 30 min.

The microsomes from pea shoots were also fractionated by aqueous polymer two-phase partition in a 10-step hand counter-current procedure [24]. The 10 systems each weighed 10.00 g and contained 6.0% (w/w) Dextran T500, 6.0% (w/w) PEG3350, 5.0 mmol·kg^-1 ^KPi, pH 7.8, and 0.25 mol·kg^-1 ^sucrose. The membrane sample was included in the first tube. After shaking and phase separation, the upper phase of the first tube was transferred to a fresh lower phase in tube number two and fresh upper phase was added to the first tube. The procedure was repeated until the original upper phase had reached tube number 10 and all tubes contained both upper and lower phase. All 10 complete two-phase systems were each diluted with 10 mM HEPES/KOH pH 7.5, 0.25 M sucrose and 10 mM KCl and pelleted twice at 100,000 × g_max_.

A membrane fraction enriched in ER was obtained from the first tube of the 10-step separation.

### Acyl incorporation and lipid analysis

Suspended pea plasma membranes, containing 25 μg protein unless otherwise stated, were incubated with 26 μM [1-^14^C]18:1-CoA (2 Gbq/mmol) or [1-^14^C]16:0-CoA (2 Gbq/mmol) in a total volume of 100 μl of 0.33 M sucrose, 30 mM HEPES/pH 7.0, 10 mM KCl. With soybean plasma membranes, the assay routinely used 100 μg protein and 2.5 mM Mg-acetate was included in the assay. Further additions were according to figure and table legends. Prior to use, dissolved lysophospholipids were dried under nitrogen to remove the organic solvent, suspended in incubation medium and sonicated for 15 min. When [^14^C]lysoPC (*sn-1 *[^14^C]16:0, 2 Gbq/mmol) was used, 0.92 nmol was mixed with 7.2 nmol of unlabelled lysoPC (egg yolk; containing predominantly 16:0 or 18:0). The standard incubation time was 30 min and the reaction was stopped by adding 980 μl of a mixture of ice cold chloroform:methanol:water (30:60:15 by vol.). After addition of 0.5 ml 1.6 M HCl and 0.5 ml CHCl_3_, the lipids were extracted [42]. An aliquot was removed for determination of total lipid radioactivity by liquid scintillation counting and the remainder was used for determination of radiolabel distribution between the lipids using thin layer chromatography and radio-scanning [16].

### Other assays and experimental designs

The following marker enzymes were assayed: cytochrome C oxidase for mitochondrial inner membrane [43], choline phosphotransferase for endoplasmic reticulum [44], and 1,3-β-glucan synthase for plasma membrane [45]. Chlorophyll was assayed for thylakoid membranes [46], and binding of a monoclonal anti-β-COP anti body, M3A5 (Sigma-Aldrich, St. Louis, US; previously validated for plants [47]), was assayed for Golgi membranes. Briefly, each fraction was dot-blotted onto a nitrocellulose membrane. Antibody incubation was performed as previously described [47] except for that M3A5 was used in a 1:200 dilution and the secondary antibody containing horseradish peroxidase conjugate (Pierce, Rockford, IL, USA) was diluted 1:4000. Chemoluminescence from the peroxidase reaction was detected on Hyper film ECL (GE-helthcare, Chalfont, UK) and intensities were quantified with Syngene Bio imaging system (Cambridge, UK). The protein concentration was measured using the bicinchoninic method [48] after solubilization of membrane fractions in 0.25% Triton X-100 for 10 min prior to addition of the protein determination reagent. SDS-PAGE, native gel electrophoresis and protein identification by MALDI-TOF or nano LC-FTISR spectroscopy (Swegene Proteomics Centre, Göteborg University, Sweden) were performed as described previously [24].

For the studies of the effects of PLA_2_, 0.001 U of bee venom PLA_2 _was used, together with 100 μM CaCl_2_. Exogenously added radiolabelled PC was *sn-1,2 *[^14^C]16:0-PC.

To observe plasma membrane-associated ER, we used *A. thaliana *transformed with GFP in the ER lumen [31]. We chose ER lumen-localized GFP (instead of ER membrane-localized GFP) to maintain a native ER membrane surface for the studies of membrane-membrane interactions. The isolated plasma membrane fraction was subjected to cycles of freezing and thawing to invert a population of the vesicles [32]. The resulting plasma membranes were visualized by using the fluorescent probe FM4-64. The confocal microscopy images were obtained as described [20] using equipment at the Centre for Cellular Imaging (Swegene, Göteborg University).

All experimental set ups were performed 2–5 times, each time using a plasma membrane fraction (or other membrane fraction) isolated from an independently cultivated plant batch. The data from one representative experiment from each set up are presented, as absolute radio-labelling varied between independent experiments. Trends or differences between treatments were consistent between independent experiments of the same design, except when noted.

## Abbreviations

16:0, hexadecanoic acid; 18:0, octadecanoic acid; 18:1, cis-9-octadecenoic acid; 16:0-CoA, hexadecanoyl-CoA; 18:1-CoA, cis-9-octadecenoyl-CoA; CoA, coenzyme A; DAG, diacylglycerol; ER, endoplasmic reticulum; FFA, free fatty acid; GFP, green fluorescent protein; MAM, mitochondria-associated membrane; PA, phosphatidic acid; PAM, plasma membrane associated membrane; PC, phosphatidylcholine; PE, phosphatidylethanolamine; PI, phosphatidylinositol; PLA_2_, phospholipase A_2_; PLAM, plastid associated membrane;

## Authors' contributions

JMK initiated the study and carried out the assays on soybean membranes. KEL and ASS designed the pea studies and KEL performed all isolations of and assays on pea membranes. HT contributed with the confocal microscopy and the Golgi antibody studies. KEL and ASS wrote the manuscript and all authors read and approved the final version.
